# A new, morphologically cryptic bush-cricket discovered on the basis of its song in the Carpathian Mountains (Insecta, Orthoptera, Tettigoniidae)

**DOI:** 10.3897/zookeys.680.12835

**Published:** 2017-06-14

**Authors:** Ionuţ Ştefan Iorgu, Elena Iulia Iorgu, Gergely Szövényi, Kirill Márk Orci

**Affiliations:** 1 “Grigore Antipa” National Museum of Natural History, Kiseleff blvd. 1, Bucharest, Romania; 2 Department of Systematic Zoology & Ecology, Eötvös Loránd University, Pázmány P. sétány 1/c, H–1117, Budapest, Hungary; 3 MTA-ELTE-MTM Ecology Research Group of the Hungarian Academy of Sciences, Eötvös Loránd University and Hungarian Natural History Museum, Pázmány P. sétány 1/c, H–1117, Budapest, Hungary

**Keywords:** Bioacoustics, Carpathians, *Isophya*, new species, taxonomy

## Abstract

A new, morphologically cryptic species of phaneropterine bush-crickets is described from the grasslands of the Romanian Eastern Carpathians. Despite the morphological and acoustic similarities with the recently described *Isophya nagyi* Szövényi, Puskás & Orci, *I. bucovinensis* **sp. n.** is characterized by a peculiar male calling song, with faster syllable repetition rate (160–220 syllables per minute, at 22–27°C) and less complex syllable structure (composed of only two elements instead of three observable in *I. nagyi*). The morphological description of the new species is supplemented with an oscillographic and spectrographic analysis of the male calling song and male–female pair-forming acoustic duet. An acoustic signal-based identification key is provided for all the presently known species of the *Isophya camptoxypha* species group, including the new species.

## Introduction

The glaciers of the Quaternary ice ages shaped distribution patterns in biodiversity and are considered as one of the primary forces of population divergence and speciation (Stewart et al. 2010; Homburg et al. 2013). The Carpathians proved to be one of the main glacial refugia for the re-colonization of northern regions of Europe in Late Glacial and Postglacial periods (Mráz and Ronikier 2016, Varga 2011). During the Last Glacial Maximum, this mountain range was mostly unglaciated, providing suitable conditions for many species of temperate fauna and flora to survive (Zasadni and Kłapyta 2014). Also, the Carpathians are an important center of biodiversity that developed a characteristic biota with a high proportion of endemic taxa, due mainly to the isolation from other mountain ranges and to its archipelago-like structure, forming a series of habitat islands (Mráz and Ronikier 2016). The Eastern and Southern Carpathians proved to be biodiversity hotspots within the Carpathian Mountains, being the richest areas in endemics regarding several groups of vascular plants and invertebrates, such as arachnids, mollusks and insects (Kenyeres et al. 2009, Bálint et al. 2011, Theissinger et al. 2013, Gajdoš et al. 2014, Cameron et al. 2016, Hurdu et al. 2016, Mráz et al. 2016). Most of these endemics are organisms with low-dispersal abilities, species likely to show stronger genetic differentiation patterns due to the absence of gene flow (Homburg et al. 2013). This appears to be also the case for the flightless bush-crickets of the *Isophya camptoxypha* species-group, which could be an interesting model to explore speciation driven by climate changes in high mountainous landscapes. These species are slowly moving brachypterous insects, occurring in cold and mesic meadows from montane to subalpine altitudes, always preferring broadleaved dicotyledonous plants: *Veratrum* sp., *Rubus* sp., *Rumex* sp., *Urtica* sp., *Aconitum* sp., *Vaccinium* sp., *Gentiana* sp. etc. (Orci et al. 2010b, Iorgu 2012, Szövényi et al. 2012).

Although the species-level identification of specimens in this species-group is difficult due to morphological uniformity, the oscillographic structure of male calling songs shows clear differences between species (Heller et al. 2004, Orci et al. 2005, Orci et al. 2010a, Iorgu 2012, Iorgu et al. 2017). In these bush-crickets, one of the main functions of acoustic signaling is to transmit information between males and females during their pair-forming behavior. Typically, males stridulate spontaneously and females decide which male to approach or to respond to acoustically (Heller 1990). In the latter case, the singing interaction of male and female forms a duet. Female song preferences and male song pattern is believed to be a significant ethological component of the species-specific mate recognition system of these insects (Orci 2007, Orci et al. 2010a, Iorgu 2012, Szövényi et al. 2012). Therefore, analyzing the acoustic signals of these bush-crickets may reveal previously unrecognized, morphologically cryptic species when studying formerly unexamined populations. Here we describe a new *Isophya* species recognized by studying its male calling song. A characterization of the basic features of its distinctive male calling song and male–female acoustic duet are presented, and a male calling song based identification key for the *Isophya camptoxypha* species-group is provided.

## Materials and methods

### Specimen collection

During the first visit in Călimani Mountains in the summer of 2012, several *Isophya* specimens were collected by visual examination of the subalpine vegetation in meadows below Pietrosul Peak (1800–1900 m asl) and at lower altitudes, near the village Gura Haitii (900–1000 m asl). These specimens turned out to be very different from all other known *Isophya* species regarding the pattern of male calling song and are described here as a new, cryptic species: *Isophya bucovinensis* sp. n. Later, we discovered this insect at other highland locations in the northern and western areas of the large volcanic caldera of Călimani Mountains and also in Suhard Mountains, in the south-western and western parts of Bucovina region.

### Morphology

The description of examined morphometric characters follows Orci et al. 2005. Measurements were taken with a digital caliper for the following morphometric variables in males: width of head, length of pronotum, frontal width of pronotum, caudal width of pronotum, length of left tegmen, width of left tegmen, length of cercus, length of hind femur.

Photos were taken with a Canon EOS 6D DSLR camera, Canon EF 180 mm f3.5L 1:1 and Canon MP–E 65 mm f2.8 5:1 macro lenses. Scanning Electron Microscope photos were taken of the stridulatory files and male cerci.

### Acoustic examination and terminology

The calling song of eleven males and the “mate searching” duet of four males and five females were recorded and analyzed. Recordings were taken with an Edirol R–09HR digital recorder (microphone frequency response 20–40000 Hz, sampling rate of 96000 Hz, 24–bit amplitude resolution). The sound analyses were run using the software Audacity 2.1.2.

The acoustic terminology follows Ragge and Reynolds (1998) and Heller et al. (2004): *calling song* – spontaneous song produced by an isolated male; *syllable* – the song produced by one opening–closing movement cycle of the tegminae; *impulse* – a simple, undivided transient train of sound waves (the highly-damped sound impulse arising as the impact of one tooth of the stridulatory file); *click* – an isolated, distinct impulse. As the calling songs of *Isophya* species are amplitude modulated signals similarly to the stridulation of most phaneropterine (Heller et al. 2015), we measured several oscillographic characters which may be useful when comparing the song of the new species with the songs of other *Isophya* species: duration of syllable (DS), duration of “B”–element (DB), duration of “C”–element (DC), number of impulses in “B”–element (NIB), number of impulses in “C”–element (NIC), silent interval between “B”– and “C”–elements (IntBC), silent interval between successive syllables (IntS), the longest impulse series in a syllable (LIS). The delay of female response was measured from the beginning of “C”–element in male syllable to the beginning of female reply (F). The detailed terminology of the measured male song characters in *I. bucovinensis* sp. n. reflects our hypothesis about the homology of these signal elements with syllable-elements of *Isophya nagyi* (see Szövényi et al. 2012), which seems to be the closest relative of *I. bucovinensis* sp. n. The first element of the syllables produced by *Isophya nagyi* is missing in the syllables of *I. bucovinensis* sp. n., therefore in the latter species the first syllable element is referred to as “B”, and the second one is termed “C”.

## Taxonomy

### Isophya bucovinensis sp. n.

Taxon classificationAnimaliaOrthopteraTettigoniidae

http://zoobank.org/797AC7E6-0702-4510-AC0C-4C0E454EF81C

Fig. 1A, B

#### Type locality.

Romania, Eastern Carpathian Mountains, Călimani Mountains.

#### Type material.

**Holotype**: male. Original label: “Romania, Suceava County, Călimani Mountains, 12 Apostoli Peak, 47.221233°N, 25.213896°E, alt. 1730 m asl, 2015.07.27, leg. I. Ș. Iorgu”, coll. “Grigore Antipa” National Museum of Natural History, Bucharest, Romania. **Paratypes**: 1 ♂ 1 ♀, labeled: “Romania, Suceava County, Gura Haitii, 47.226236°N, 25.300690°E, alt. 990 m asl, 2012.06.21, leg. I. Ș. Iorgu”; 2 ♂♂ 2 ♀♀, labeled: “Romania, Suceava County, Călimani Mountains, meadow below Pietrosul Peak, 47.130767°N, 25.191679°E, alt. 1800 m asl, 2012.07.20, leg. I. Ș. Iorgu”; 1 ♂ 1 ♀, labeled: “Romania, Suceava County, Suhard Mountains, Omu Peak, 47.507519°N, 25.090278°E, alt. 1860 m asl, 2013.07.21, leg. I. Ș. Iorgu”; 2 ♂♂ 2 ♀♀, labeled: “Romania, Suceava County, Călimani Mountains, 12 Apostoli Peak, 47.221233°N, 25.213896°E, alt. 1730 m asl, 2015.07.27, leg. I. Ș. Iorgu”; 2 ♀♀, labeled: “Romania, Suceava County, Călimani Mountains, 12 Apostoli Peak, 47.222835°N, 25.212571°E, alt. 1730 m asl, 2016.09.03, leg. I. Ș. Iorgu”, all in coll. “Grigore Antipa” National Museum of Natural History, Bucharest, Romania; 3 ♂♂ 3 ♀♀, labeled: “Romania, Suceava County, Călimani Mountains, 12 Apostoli Peak, 47.221233°N, 25.213896°E, alt. 1730 m asl, 2015.07.27, leg. I. Ș. Iorgu”, coll. Hungarian Natural History Museum, Budapest, Hungary.

**Audio recordings**: 1 ♂, Gura Haitii, 2012.06.21 (air temperature 25°C); 1 ♂, meadow below Pietrosul Peak, Călimani Mts., 2012.07.20 (22°C); 2 ♂♂ 3 ♀♀, meadow below Pietrosul Peak, Călimani Mts., 2012.07.20 (27°C); 3 ♂♂, Omu Peak, Suhard Mts., 2013.07.21 (25°C); 4 ♂♂ 2 ♀♀, 12 Apostoli Peak, Călimani Mts., 2015.07.27 (24°C).

**Figure 1.**
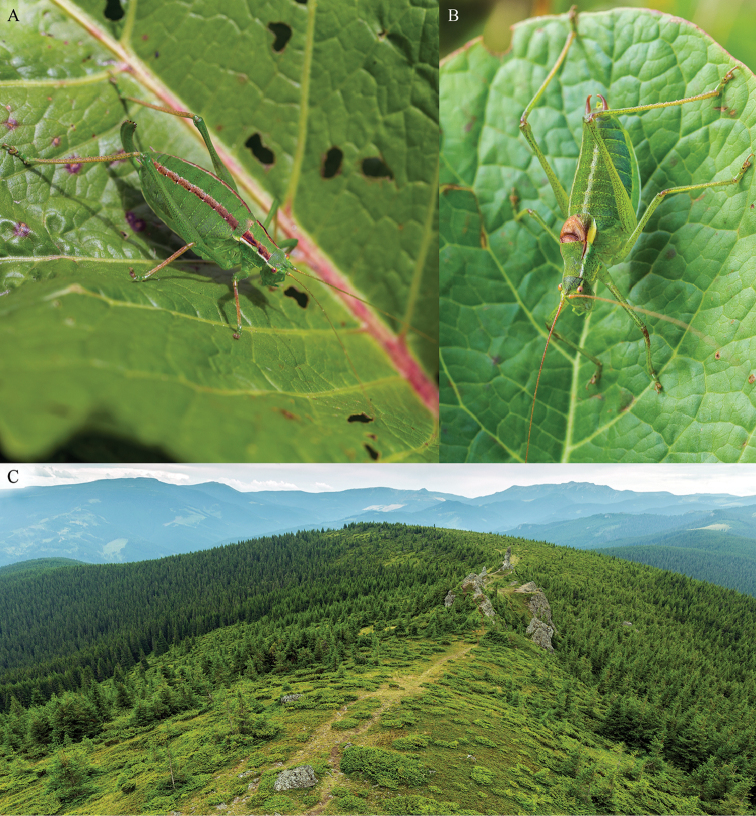
*Isophya bucovinensis* sp. n.: **A** female habitus **B** male habitus **C** habitat in Călimani Mountains, at 12 Apostoli Peak (1730 m asl) on 27 July 2015.

#### Other material.

*Isophya bucovinensis* sp. n.: 1 ♂ 1 ♀, Romania, Suceava County, Călimani Mountains, 47.208928°N, 25.200489°E, alt. 1640 m asl, 2015.07.27, leg. I. Ș. Iorgu; 2 ♂♂ 3 ♀♀, Romania, Suceava County, Călimani Mountains, 47.202448°N, 25.197817°E, alt. 1620 m asl, 2015.07.28, leg. I. Ș. Iorgu; 1 ♂ 4 ♀♀, Romania, Suceava County, Călimani Mountains, 47.134651°N, 25.178858°E, alt. 1680 m asl, 2016.08.07, leg. I. Ș. Iorgu. *Isophya nagyi*: 10 ♂♂ 2 ♀♀ (audio recorded at 21.2–24°C), Romania, Suceava County, Călimani Mountains, 47.137°N, 25.256°E, alt. 1340 m asl and 47.141°N, 25.267°E, alt. 1580 m asl, 2011.07.27, leg. K. M. Orci, G. Puskás & G. Szövényi.

#### Diagnosis.

Fastigium verticis approximately half as wide as scapus, male elytra short and narrow, slightly shorter than or as long as pronotum, reaching anterior part of second abdominal tergite. Length of cubital vein 3/4 of the width of pronotum posterior margin, forming an obtuse angle of nearly 110° at the right margin of left tegmen. The stridulatory file is 2.2–2.5 mm long and contains 105–130 pegs. Male cerci slender, 2.4–2.7 mm long, gradually and moderately incurved in distal 1/4, tapering in an apical denticle. Ovipositor short, upcurved, 8.3–9.2 mm long. Male calling song consists of a long sequence of two-component syllables (Fig. 3A, B) repeated at a rate of 160–220 per minute (at 22–27°C air temperature). Each syllable lasts for 237–385 ms and is formed of two parts: a longer group of 46–79 impulses (156–286 ms) shortly followed by a higher amplitude group of 2–7 clicks (3–28 ms) (Fig. 3C, D). Females reply right after the male syllable, with a latency of 48–66 ms (Fig. 4C). All these sounds are produced by closing the tegmina.

Morphologically, *Isophya bucovinensis* sp. n. belongs to the *Isophya camptoxypha* species group. Within this complex, *I. bucovinensis* sp. n. and *I. nagyi* differ from all the other species in the relatively high number of pegs observable in the male stridulatory file. Furthermore, *Isophya bucovinensis* sp. n. can be easily distinguished from *I. nagyi* by examining the number of impulse groups within a syllable (three in *I. nagyi* vs. two in *I. bucovinensis* sp. n.) and the syllable repetition rate during continuous singing: 60–80 syllables/minute in *I. nagyi* (21–24°C) and 160–220 in *I. bucovinensis* sp. n. (22–27°C) (Fig. 4).

#### Description.

**Figure 2.**
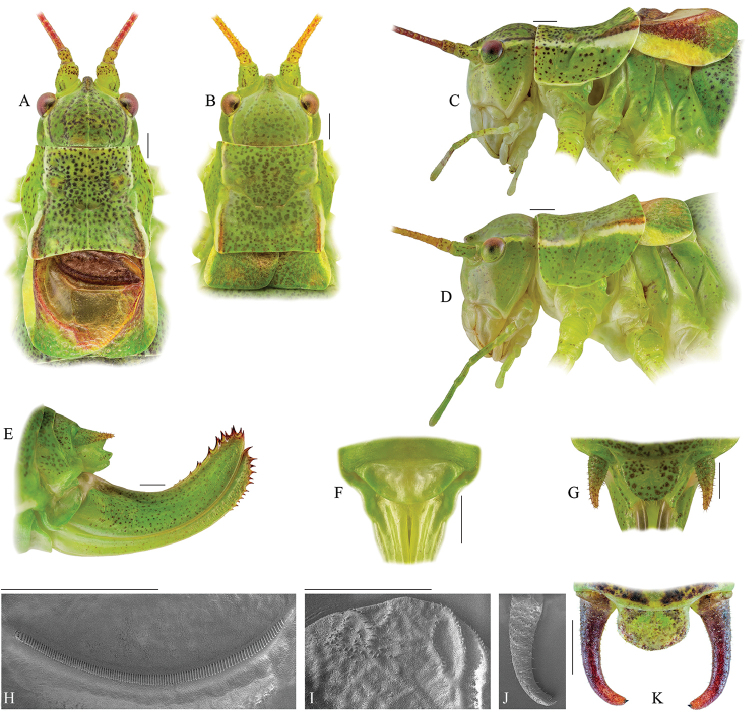
Photographs of the typical eidonomy of *Isophya bucovinensis* sp. n. male (**A, C, H, J, K**) and female (**B, D, E, F, G, I**). **A, B** head, pronotum and tegmina in dorsal view **C D** head, pronotum and tegmina in lateral view **E** ovipositor in lateral view **F** female subgenital plate **G** female cerci in dorsal view **H** male stridulatory file (SEM) **I** female stridulatory bristles (SEM) **J** male cercus (SEM) **K** male cerci in dorsal view. Scale bars 1 mm.

***Male*** (Fig. 2A, C, H, J, K; Table 1). Fastigium verticis half as wide as scapus, slightly tapering frontward. Head width 3.1–3.7 mm, 1.3 times the maximum pronotum width. The pronotum is 3.9–5 mm long, with lateral carinae nearly parallel in prozona, broken at traverse sulcus, widen and divergent in metazona. Saddle–shaped from a lateral view, the paranota with concave dorsal margin; anterior and ventral borders straight, posterior edge moderately convex. Tegmina short and narrow, slightly shorter than or as long as pronotum, reaching the second abdominal tergite. Venation reticulate, Cu2 vein swollen, its length 3/4 of pronotum posterior margin. The angle between cubital veins of approximately 70–80°, speculum large and quadrangular. At the distal end of Cu2, the right margin of left tegmen forms an obtuse angle of nearly 110°. Stridulatory file arcuate, 2.2–2.5 mm long, with 105–130 teeth. Hind femur without ventral spines, 3.5–4 times long as pronotum. Epiproct almost twice as wide as long. Cerci covered by fine hairs, slender, 2.4–2.7 mm long, gradually narrowing towards tip, slightly curved in apical fourth. Terminal denticle strong, located in middle of cercus apex. Subgenital plate moderately elongated, narrowed apically with triangular apical incision and more or less acute lobes on its caudal margin.

**Table 1.** Morphometric characters measured in males of *Isophya bucovinensis* sp. n. (N = 10) and *I. nagyi* (N = 20) (values in millimeters).

Morphometric parameters	*Isophya bucovinensis* sp. n.			*Isophya nagyi*		
	Min	Max	Mean ± SD	Min	Max	Mean ± SD
Width of head	3.1	3.7	3.37 ± 0.213	3.4	3.8	3.64 ± 0.105
Length of pronotum	3.8	4.3	4.02 ± 0.198	3.3	4.3	3.84 ± 0.261
Frontal width of pronotum	3.2	3.4	3.32 ± 0.083	3	3.6	3.19 ± 0.159
Caudal width of pronotum	3.9	5.0	4.41 ± 0.442	4.4	5.2	4.6 ± 0.201
Length of left tegmina	3.4	4.8	3.88 ± 0.449	4	4.8	4.44 ± 0.211
Width of left tegmina	3.1	4.2	3.7 ± 0.361	3.8	4.4	4.1 ± 0.16
Length of hind femur	13.5	15.9	14.7 ± 0.718	14.3	16.8	15.58 ± 0.747
Length of cercus	2.4	2.7	2.5 ± 0.103	2.2	2.8	2.54 ± 0.131

Coloration green, densely punctuated with fine, dark green and brown spots. Some specimens with two dorsal, parallel, bilateral stripes from pronotum to end of abdomen, red, orange, or yellow colored. Antennae greenish, reddish or brownish, compound eyes bicolored: upper part brown or red and lower part green. Disc of pronotum green, bordered by a yellowish or whitish band, from behind the eye to the posterolateral angle of wing, complemented by a reddish brown stripe in metazona. Tegmina brown or dark red, with green margins and greenish or yellowish white costal margin, as continuation of pronotum band. Legs usually green, brownish or reddish. Cerci brown or reddish brown.

***Female*** (Fig. 2B, D, E, F, G, I). Head roughly as in male. Pronotum disc marginally widening in its posterior part, with lateral carinae straight in prozona. Paranota similar to these of male. Tegmina with dense reticulate venation, approximately 1/4–1/3 the length of pronotum, reaching the first abdominal tergite, with more or less rounded margins. Right tegmen with two dorsal fields of stridulatory bristles on cubital veins. Epiproct semicircular. Cercus short, hairy, conical. Subgenital plate rounded, narrow. Ovipositor relatively short, upcurved, 8.3–9.2 mm long, with 8–9 spines on dorsal margin and 7–9 spines on ventral margin, gonangulum ellipsoid.

Body coloration similar to that of male. Tegmina green with brownish inner and whitish or yellowish lateral edges. Ovipositor green with dark brown spines.

#### Bioacoustics

**Figure 3.**
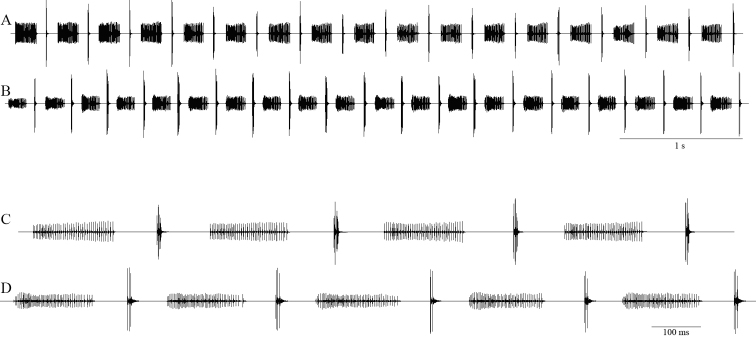
Oscillographic representation of the calling songs (**A, B**) of two males of *Isophya bucovinensis* sp. n. and detailed syllables (**C, D**). **A, C** Călimani Mountains, 12 Apostoli peak, 1730 m asl (air temperature 24°C) **B, D** Suhard Mountains, Omu peak, 1860 m asl (air temperature 25°C).

**Figure 4.**
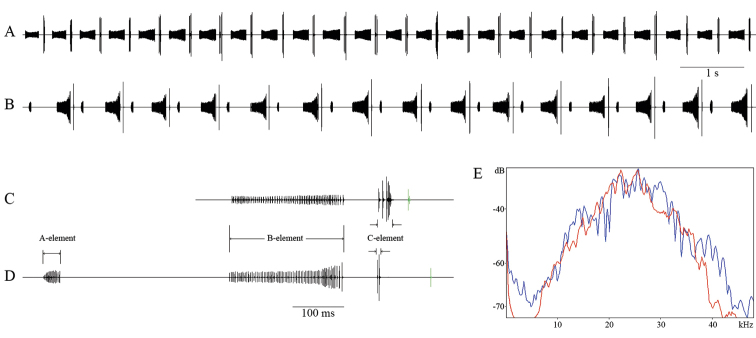
Acoustic signals analysis in two close–related species: **A, B** oscillographic pattern of male calling song **C, D** a detailed syllable and the female response (marked with green) **E** spectrum (function: Hanning window, size: 512). **A, C, E** red line *Isophya bucovinensis* sp. n. **B, D, E** blue line *Isophya nagyi*.

(Figs 3, 4; Table 2). Males stridulate in the evening and at night. Typically, the song consists of series of 8–35 syllables; uninterrupted series of more than 200 syllables were also recorded. Syllable repetition rate is 160–220 syllables/minute at 22–27°C. *Isophya bucovinensis* sp. n. produces a single syllable type, lasting for 237–385 ms (mean ± SD: 311.6 ± 57.85 ms, n = 11 males) and formed of two elements: the main part (“B”) is a longer group of 46–79 impulses (mean ± SD: 60.1 ± 11.54), 156–286 ms (mean ± SD: 215.2 ± 52.7 ms) long, shortly followed by the terminal “C” part, a higher amplitude group of 2–7 impulses (mean ± SD: 4.4 ± 1.64), 3–28 ms (mean ± SD: 16.45 ± 7.39 ms) long. The same impulse interval was measured in both “B” and “C” groups, 2–4 ms. The two parts are separated by a short silent interval of 59–94 ms (mean ± SD: 76.85 ± 10.56 ms). The following syllable begins 76–125 ms (mean ± SD: 94.2 ± 17 ms) later (Fig. 3). All sounds are produced when the male closes the tegmina. Apart from the described typical syllable structure, some males produce syllables without the “C” element at the beginning of syllable series. The calling song’s dominant frequency components are between 10–35 kHz, highest peak at approximately 25 kHz (Fig. 4E).

**Table 2.** Descriptive statistics for oscillographic parameters of the male calling song and for female response delay in *Isophya bucovinensis* sp. n. and *I. nagyi* (duration values in milliseconds). Number of specimens for which measurements were made: *I. bucovinensis* 11 males, 5 females; *I. nagyi* 10 males, 2 females.

Calling song parameters	*Isophya bucovinensis* sp. n.			*Isophya nagyi*		
	Min	Max	Mean ± SD	Min	Max	Mean ± SD
Duration of syllable (DS)	237	385	311.6 ± 57.85	489.6	934.3	727.33 ± 148.27
Duration of “B”–element (DB)	156	286	215.2 ± 52.7	129.5	217.8	188.96 ± 32.03
Number of impulses in “B”–element (NIB)	46	79	60.1 ± 11.54	38.7	79	56.83 ± 12.77
Silent interval between “B”– and “C”–elements (IntBC)	59	94	76.85 ± 10.56	54.2	91.5	69.43 ± 12.36
Duration of “C”–element (DC)	3	28	16.45 ± 7.39	1	5.4	3.59 ± 1.62
Number of impulses in “C”–element (NIC)	2	7	4.4 ± 1.64	1	4	2.6 ± 0.96
Silent interval between successive syllables (IntS)	76	125	94.2 ± 17	121.2	227.9	160.24 ± 34.24
Female response delay (F)	48	66	58.7 ± 4.77	71	127	112.2

Females find males by phonotaxis and emit simple clicks if the male song is attractive for them. In the resulting male–female acoustic duet, the female replies only after the male syllables (“C”–part), with a latency of 48–66 ms (mean ± SD: 58.7 ± 4.77 ms, n = 200 replies from 5 females) (Fig. 4C).

#### Etymology.

The specific name is derived from the historical region of Bucovina, “Land of the beech forests”.

#### Distribution and natural history.

The new species populates mesophytic montane meadows in the south–western part of Bucovina, within the altitudinal range of 900–1900 m (Fig. 1C). The insects were collected from broad leaves of various dicotyledonous plants: *Veratrum* sp., *Rumex* sp., *Rubus* sp., *Aconitum* sp., *Vaccinium* sp., *Urtica* sp., *Gentiana* sp., *Hypericum* sp. etc. The distribution of this bush-cricket includes highlands from northern and western Călimani Mountains, the northern part of Suhard Mountains (Eastern Carpathians) (Fig. 6) and most likely occurs also in Dorna depression and the central and southern parts of Suhard Mountains.

#### Conservation status.

*Isophya bucovinensis* sp. n. is known only from Călimani and Suhard Mountains. It could be considered as *Endangered B1ab(iii, v)+2ab(iii, v)* following IUCN’s Red List categories and criteria version 3.1 (known EOO=234 km^2^ and AOO=28 km^2^; the constant degradation of species’ habitat is the result of mechanized mowing in June and July, and overgrazing, especially in Suhard highlands) (IUCN 2012).

#### Discussion.

In this study, we report the discovery and give the description of a new bush-cricket species recognized based on its distinctive calling song. The basic pattern of its song is not unique within the genus *Isophya*: a series of evenly repeated syllables, each consisting of two elements: a longer impulse series followed by a short impulse group (see e. g. Heller 1988, Chobanov et al. 2013). However, species producing this male song pattern can be found in several different, morphology-based species-groups (Warchałowska-Śliwa et al. 2008, Chobanov et al. 2013). One of these species-groups is the *Isophya camptoxypha* complex. Six morphologically closely-related species inhabiting the Carpathians were recently placed within this group: *I. camptoxypha* Fieber, *I. ciucasi* Iorgu & Iorgu, *I. nagyi*, *I. sicula* Orci, Szövényi & Nagy, *I. posthumoidalis* Bazyluk and *Isophya dochia* Iorgu (Szövényi et al. 2012, Iorgu 2012). Because of its similar morphology, *I. bucovinensis* sp. n. also belongs to this group.

Since the species of the *Isophya camptoxypha* species-group are all very similar to each other regarding the morphology, we believe that a male calling song based identification key will be helpful to identify specimens from populations of unknown species identity.

Identification key for the presently recognized species of *I. camptoxypha* species group, based on male song characters:

**Table:** 

1	Male calling song consists of syllable groups separated from each other by a characteristic interval. Syllable groups are composed of two syllable types (“A” and “B”), each group being a series of “A” syllables followed by one “B” syllable containing conspicuous, high amplitude clicks (Fig. 5A, B)	**2**
–	Male calling song is a series of syllables, no regular syllable groups are observable, syllables show the same basic structure (i.e. the song is composed of one syllable type) (Fig. 5C–G)	**3**
2	“A” syllables are shorter than 25 ms, the conspicuous click(s) follow(s) a silent interval of 300–450 ms (Fig. 5A)	***I. posthumoidalis***
–	“A” syllables have a duration of 30–60 ms, the high amplitude click(s) between the syllable groups follow(s) a 550–950 ms long silent interval (Fig. 5B)	***I. dochia***
3	The longest impulse series in the syllables (LIS) is shorter than 80 ms	**4**
–	LIS is longer than 100 ms	**5**
4	LIS falls between 25–60 ms, consisting of more than 10 impulses (Fig. 5C)	***I. camptoxypha***
–	LIS is shorter than 25 ms, consisting of 1–3 impulse(s) (Fig. 5D)	***I. sicula***
5	Syllables consisting of two impulse groups	**6**
–	Syllables consisting of three impulse groups (Fig. 5F)	***I. nagyi***
6	Interval after LIS is longer than 300 ms (Fig. 5E)	***I. ciucasi***
–	Interval after LIS is shorter than 150 ms (Fig. 5G)	***I. bucovinensis* sp. n.**

**Figure 5.**
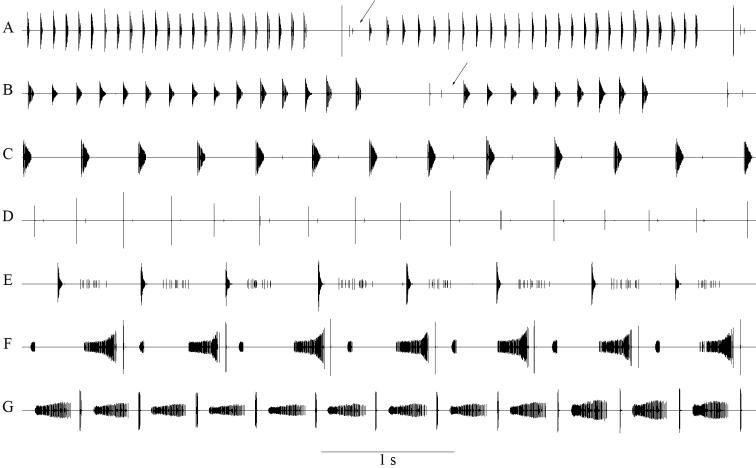
Oscillograms showing the typical pattern of male calling songs in each species of the *Isophya camptoxypha* species-group: **A** *I. posthumoidalis* (25°C, Bieszczady Mountains, Poland) **B** *I. dochia* (24.8°C, Gurghiu Mountains, Romania) **C** *I. camptoxypha* (25°C, Bieszczady Mountains, Poland) **D** *I. sicula* (24.8°C, Harghita-Ciceu Mountains, Romania) **E** *I. ciucasi* (25°C, Ciucaș Mountains, Romania) **F** *I. nagyi* (22.5°C, Călimani Mountains, Romania) **G** *I. bucovinensis* sp. n. (25°C, Suhard Mountains, Romania). Time scale (shown at the bottom) is the same for all oscillograms in the figure. The arrows indicate the characteristic interval separating syllable groups.

The duration values given in the above key are based on male songs recorded at an ambient air temperature between 20–25°C. To identify specimens, a song recording should be made in that range of air temperature. The key is most reliable when mean values based on at least 5–10 measurements per specimen are used in all quantitative characters.

The easiest way to identify male specimens of *Isophya bucovinensis* sp. n. is to examine both the male stridulatory file and the pattern of male calling song. Within the *I. camptoxypha* species group, only two species have male stridulatory files containing more than 100 stridulatory pegs: *I. nagyi* and *I. bucovinensis* sp. n. Interestingly, except for the different number of syllabic elements and syllable repetition rates, the syllables produced by males of *Isophya bucovinensis* sp. n. and *I. nagyi* show remarkable similarities in oscillographic structure (Fig. 4). The longest impulse series of their syllables (element “B”) are almost identical (156–286 ms, 46–79 impulses in *I. bucovinensis* sp. n. and 129–217 ms, 38–79 impulses in *I. nagyi*), and also the high amplitude, short impulse group (element “C”) after the long impulse series is rather similar in the two species (3–28 ms, 2–7 impulses in *I. bucovinensis* sp. n. and 1–6 ms, 1–4 impulses in *I. nagyi*). Because of these similarities in song and due to the high level of morphological similarity, we hypothesize that the two species are likely to be a pair of young sister species. This premise should be examined during subsequent studies relying on molecular genetic analysis. Spectral properties of the male calling songs are similar in these two species, most likely due to the comparable dimensions of male stridulatory apparatus (Fig. 4E).

In *Isophya bucovinensis* sp. n., the delay of female response is much shorter (48–66 ms) than that in *I. nagyi* (71–127 ms) (Fig. 4C, D). Since female reply delay proved to be a critical parameter for males to recognize female response as a conspecific signal in a number of phaneropterine bush-crickets (Heller & von Helversen, 1986), the detected difference in response delay is likely to make the responses of *I. bucovinensis* sp. n. females ineffective for communicating with *I. nagyi* males and vice versa. Also, another impediment for heterospecific communication in these two species is that the responses of *I. nagyi* females would overlap with a dense impulse series of the male’s next syllable if she would try to duet with a male *I. bucovinensis* sp. n.; the detection of the female signal is less likely if the male’s own signal masks it. Therefore, in addition to the clear difference of male songs, the divergence of female response delay also suggests that the two taxa are best treated as specifically distinct. However, several interesting questions arise regarding the male–female communication of these two species. Presently, it is uncertain which feature of the male signal serves as a marker to start the timing of female response. Females of duetting Phaneropterinae bush-crickets usually use a conspicuous motif of the male song as a response timing marker (Heller and von Helversen 1986, Heller 1990, Dobler et al. 1994). Clearly detectable features of the male song in *I. bucovinensis* sp. n. are the beginning and end of “B” and “C” elements. Among these, we consider the beginning of “C” element to be most likely the female response timing marker (this is why we measured female response delay from that male song feature). However, it is only a presumption and a future laboratory test with playback experiments applying modified male syllables will be needed to answer this question.

**Figure 6.**
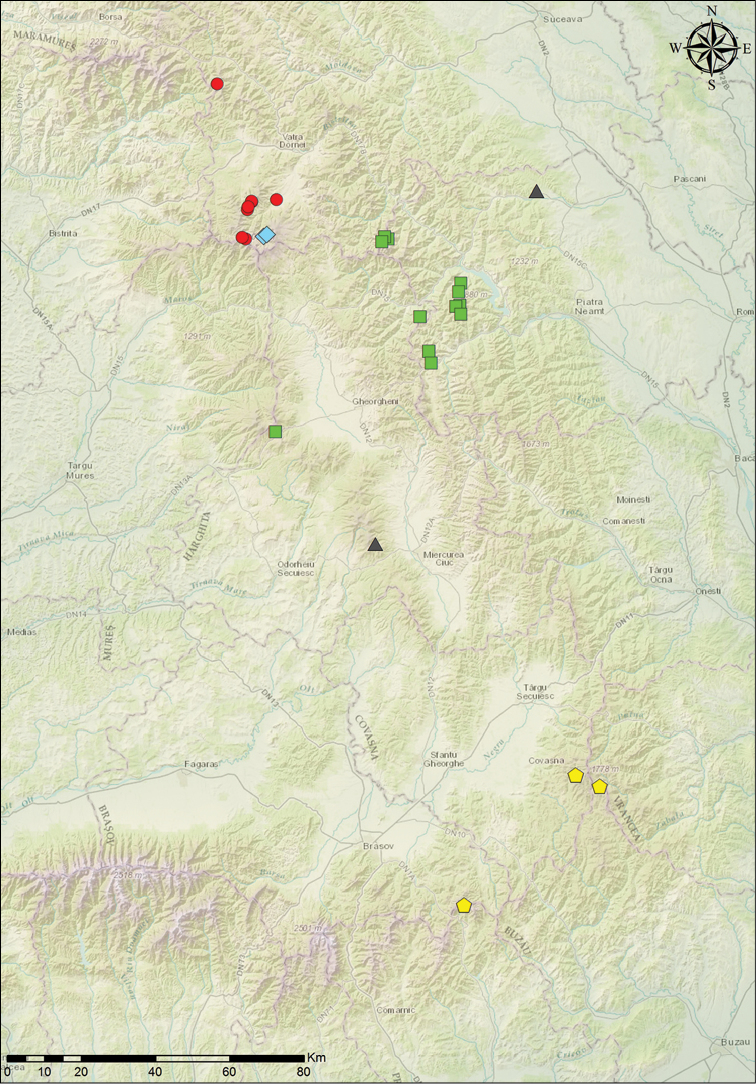
Geographic distribution of the Eastern Carpathian endemic *Isophya* species: red dots – *I. bucovinensis* sp. n., yellow pentagons – *I. ciucasi*, green squares – *I. dochia*, blue diamonds – *I. nagyi*, black triangles – *I. sicula*.

The *Isophya camptoxypha* species-group shows a surprisingly high rate of endemism in the Eastern Carpathians. Five of its recently described species have very small distribution areas (Fig. 6) : *I. ciucasi* populates montane meadows in the southern part of the Eastern Carpathians: in Ciucaș and Vrancea Mountains, *I. dochia* is known to occur in Ceahlău, Bistriței and Gurghiu Mountains, *I. sicula* is distributed in Harghita-Mădăraș and Stânișoarei Mountains, *I. nagyi* occurs in small areas in the southern part of Călimani volcanic caldera and *I. bucovinensis* sp. n. is known from the northern and western parts of Călimani, reaching Suhard mountains in north. The ancestors of these species presumably penetrated from the distribution center of this genus in Balkan–Asia Minor along the western part of Southern Carpathians and Apuseni Mountains dispersal routes (Kenyeres et al. 2009), and speciated during their expansion by isolation of the populations in the mountain chains of Eastern Carpathians.

## Supplementary Material

XML Treatment for Isophya bucovinensis
